# Socioeconomic Factors and Adherence to CPAP: The Population-Based Course of Disease in Patients Reported to the Swedish CPAP Oxygen and Ventilator Registry Study

**DOI:** 10.1016/j.chest.2021.04.064

**Published:** 2021-05-08

**Authors:** Andreas Palm, Ludger Grote, Jenny Theorell-Haglöw, Mirjam Ljunggren, Josefin Sundh, Bengt Midgren, Magnus Ekström

**Affiliations:** a Department of Medical Sciences, Respiratory, Allergy and Sleep Research, Uppsala University, Uppsala, Sweden; b Centre for Research and Development, Uppsala University, Region of Gävleborg, Gävle Hospital, Gävle, Sweden; c Centre for Sleep and Wakefulness Disorders, Sahlgrenska Academy, Gothenburg University, Gothenburg, Sweden; d Department of Respiratory Medicine, School of Medical Sciences, Faculty of Medicine and Health, Örebro University, Örebro, Sweden; e Division of Respiratory Medicine & Allergology, Department of Clinical Sciences, Lund University, Lund, Sweden

**Keywords:** adherence, CPAP, OSA, socioeconomic factors, AHI, apnea-hypopnea index, ESS, Epworth Sleepiness Scale

## Abstract

**Background:**

Early identification of poor adherence to CPAP treatment is of major clinical importance to optimize treatment outcomes in patients with OSA.

**Research Question:**

How do socioeconomic factors influence CPAP adherence?

**Study Design and Methods:**

Nationwide, population-based cohort study of patients with OSA receiving CPAP treatment reported to the Swedish quality registry Swedevox between 2010 and 2018 was cross-linked with individual socioeconomic data from Statistics Sweden. Socioeconomic factors associated with CPAP adherence were identified using a multivariate linear regression model, adjusted for age and sex.

**Results:**

In total, 20,521 patients were included: 70.7% men; mean age ± SD, 57.8 ± 12.2 years; BMI, 32.0 ± 6.1 kg/m^2^; apnea-hypopnea index, 36.9 ± 22.1; Epworth Sleepiness Scale, 10.4 ± 5.0; and median nocturnal CPAP use, 355 min (interquartile range, 240-420 min). Adherence after 1.3 ± 0.8 years of CPAP use was significantly (all *P* < .001) associated with civil status (married vs unmarried: +20.5 min/night), education level (high, ≥ 13 years vs low, ≤ 9 years: +13.2 min/night), total household income (highest/third/second vs lowest quartile: +15.9 min/night, +10.4 min/night, and +6.1 min/night, respectively), and country of birth (born in Sweden with one native parent/born in Sweden with two native parents vs being born abroad: +29.0 min/night and +29.3 min/night, respectively).

**Interpretation:**

Civil status, educational level, household income, and foreign background predict CPAP adherence in a clinically significant manner and should be considered when treating OSA with CPAP.

OSA with excessive daytime sleepiness is common, affecting at least 6% of men and 4% of women,^1^ and is associated with increased risk of cardiovascular mortality and morbidity.^2^^,^^3^ CPAP treatment improves daytime sleepiness and daily functioning,^4^ mitigates an elevated risk of motor vehicle accidents,^5^ and reduces BP.^6^ In observational studies, CPAP has been shown to improve cardiovascular outcomes.^7^^,^^8^ However, this association was not shown in intention-to-treat analyses of randomized controlled trials,^9^, ^10^, ^11^, ^12^ but rather in the subgroup of patients with high adherence to CPAP.^10^, ^11^, ^12^ Four hours of mean nightly CPAP use have been identified as the cutoff point for the above-mentioned beneficial CPAP effects.

Adherence to CPAP treatment often is insufficient and a major clinical problem. As many as 29% to 83% of patients with OSA who are receiving CPAP treatment have a nocturnal CPAP use of less than 4 h.^13^ Excessive daytime sleepiness and a high apnea-hypopnea index (AHI), indicating more severe OSA, are associated with better adherence to CPAP therapy.^14^ Only a handful of studies, many of those small and with short follow-up duration, have evaluated the association between socioeconomic factors and adherence. Income,^15^^,^^16^ educational level,^17^ socioeconomic status in neighborhood,^16^, ^17^, ^18^ number of household members, and civil status^18^^,^^19^ have been associated with adherence in ,some but not all, studies.^20^, ^21^, ^22^, ^23^ The aim of this large population-based study with extended follow-up was to evaluate the association between socioeconomic factors and long-term adherence to CPAP in patients with OSA.

## Methods

### Study Design and Population

The study was an analysis of the CPAP subcohort in the prospective, longitudinal cohort study Course of Disease in Patients Reported to the Swedish CPAP Oxygen and Ventilator Registry. A detailed description of the study protocol was published previously.^24^ Patients with OSA treated with CPAP reported to the Swedevox registry between July 1, 2010, and March 12, 2018, were included, and data were cross-linked with several other quality and governmental registries. In this study, socioeconomic data from Statistics Sweden were used.

**Figure 1:**
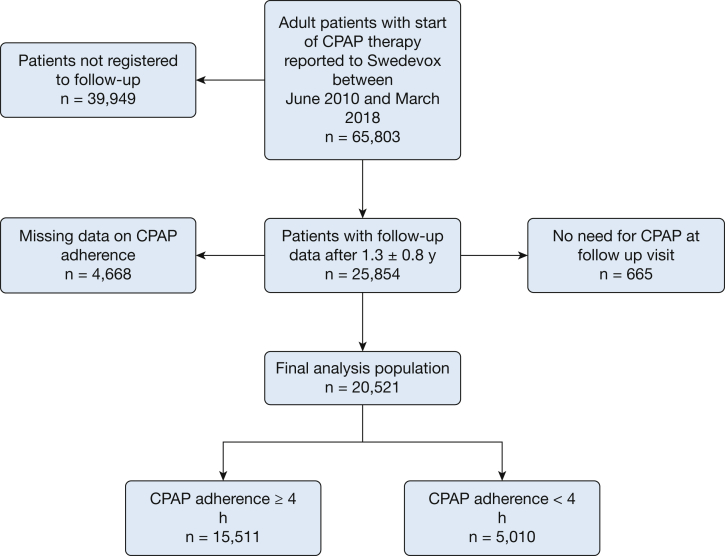
Study flowchart.

The total cohort comprised 66,265 patients, and those with complete data regarding CPAP adherence at the scheduled 1-year follow-up visit were analyzed further (n = 20,521) (Fig 1). Patients lacking reported data on nocturnal CPAP use or who claimed no further need of CPAP at the follow-up visit were excluded from subsequent analysis. Potential explanation for ceased need for CPAP can be significant weight loss with decreased symptoms of OSA or other successful sleep hygienic intervention.

The procedure for reporting to the Swedevox registry has been detailed elsewhere.^25^ In brief, CPAP-related data were reported manually to a web-based case report format by 39 sleep centers. The geographical coverage is estimated to be 90% (www.ucr.uu.se/swedevox/rapporter/arsrapporter) (e-Fig 1). Since 2015, up to 17 centers reported data via automated data transfer from the Swedish Sleep Apnea Registry (www.sesar.se). Information about sex, age, height, weight, AHI, oxygen desaturation index, excessive daytime sleepiness using the Epworth Sleepiness Scale (ESS) score,^26^ and the presence of hypertension as well as information about the use of a humidifier were reported to the registry when CPAP therapy was initiated. At follow-up, data regarding nocturnal CPAP use time (hours per total number of nights) from the CPAP’s data log were reported.

### Socioeconomic Data From Statistics Sweden

Individual data on civil status and country of birth were based on data from the nationwide Swedish Civil Registry supplied to Statistics Sweden, a government-based agency that brings official statistics to the public (www.scb.se/en). Civil status was categorized as: married or in a civil partnership, unmarried, divorced, and widow or widower. National origin was categorized as: born in Sweden with two native parents, born in Sweden with one native and one foreign parent, born in Sweden with two foreign parents, and born abroad.

Total household income at year of inclusion in the Swedevox registry or control group was obtained from the Swedish Tax Agency and was index-linked and categorized into quantiles.^27^^,^^28^ The Swedish Longitudinal Integrated Database for Health Insurance and Labour Market Studies provided data on length of education.^29^ Education was categorized into three levels: low (≤ 9 years), medium (10-12 years), and high (≥ 13 years), corresponding to compulsory school, secondary school, and postsecondary school (college and university), respectively.

### Ethics

The study was approved by the Ethical Board of Lund University (Identifier: Log No. 2018/51). Reporting to a National Quality Registry in Sweden requires careful information and verbal consent, but does not require written informed consent.

### Statistical Analyses

**Figure 2:**
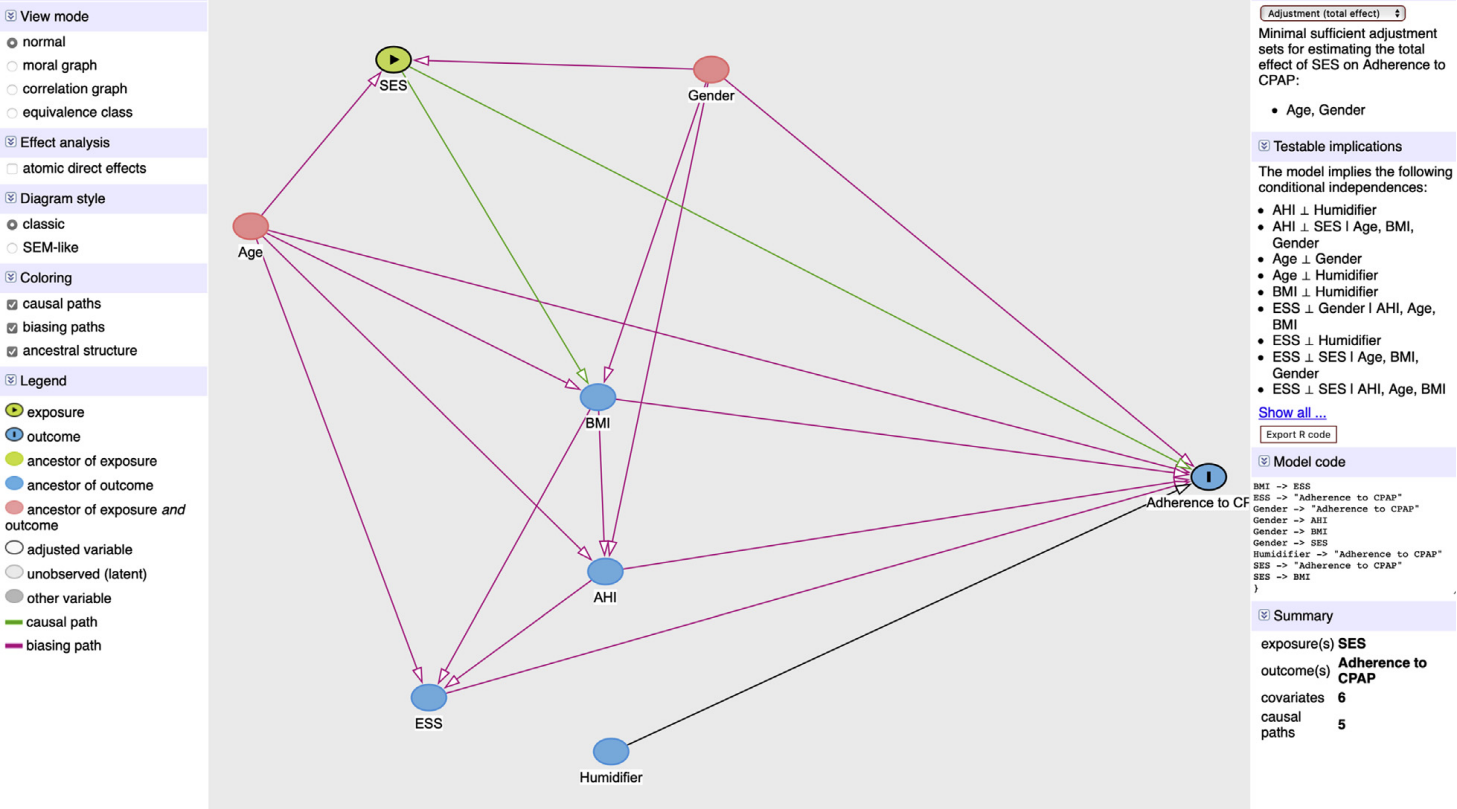
Direct acyclic graph identifying main confounders among the covariates. Dependent variable is nocturnal CPAP use time. AHI = apnea-hypopnea index; ESS = Epworth Sleepiness Scale; SES = socioeconomic status.

Normal distributed continuous data were expressed as mean ± SD, and skewed distributed continuous data were expressed as median with interquartile range. Categorical data were presented as frequencies and percentages. The *t* test was used for comparisons of continuous variables, and the χ ^2^ test was used for comparisons of categorical variables. The associations between adherence to CPAP therapy as a dependent variable and covariates were evaluated in multivariate linear and logistic regression models. Direct acyclic graphs were created using the browser-based environment DAGitty (www.dagitty.net)^30^ and identified age and sex as the main confounding factors (Fig 2). In the fully adjusted linear regression model all covariates (socioeconomic factors, age, sex, BMI, AHI, ESS score, and use of humidifier) were included to make effect sizes comparable and interpretable in a clinical context. To make a comparison of effect size between classic variables associated with CPAP adherence and socioeconomic variables possible and interpretable in a clinical context, the continuous variables age, BMI, AHI, and ESS score were transformed to categorical variables using widely accepted clinical severity thresholds. Age was stratified into young (< 40 years), middle-aged (40-< 60 years), and elderly (≥ 60 years) and total household income was stratified into quartiles to make the variable understandable for international readers. A sensitivity analysis was conducted comparing counties with reported follow-up data on more than 50% of patients with counties reporting lower follow-up rates. A *P* value of < .05 was considered statistically significant. Statistical analyses were conducted using Stata version 16.0 software (StataCorp LP).

## Results

In total, 20,521 patients were included in the analysis after a mean of 1.3 ± 0.8 years of CPAP use; 70.7% were men with a mean age of 57.8 ± 12.2 years, BMI of 32.0 ± 6.1 kg/m^2^, AHI of 36.9 ± 22.1 events/h, and ESS score of 10.4 ± 5.0. Nocturnal CPAP use of ≥ 4 h was reported in 15,511 patients (76%), whereas lower adherence was reported in 5,010 patients (Table 1, Fig 1). The median nocturnal CPAP use time was 355 min (interquartile range, 240-420 min). CPAP-adherent patients were slightly older, showed higher AHI levels at baseline, and showed a slightly lower BMI. Patients nonadherent to CPAP were less frequently married, had lower levels of education, and more frequently had a foreign background.

**Table 1:** Baseline Characteristics

Characteristic	All	Fully Adherent to CPAP (≥ 4 h/night)	Nocturnal CPAP Use < 4 h/night
No. of patients	20,521	15,511	5,010
Men	14,501 (70.7)	11,029 (71.1)	3,472 (69.3)
Nocturnal CPAP use, min	355 (240-420)	386 (328-432)	69 (0-180)
Age, y	57.8 ± 12.2	58.2 ± 11.9	56.6 ± 12.7
<40	1,632 (8.0)	1,137 (7.3)	495 (9.9)
40-60	8,938 (43.6)	6,604 (42.6)	2,334 (46.6)
≥60	9,951 (48.5)	7,770 (50.1)	2,181 (53.5)
BMI, kg/m^2^	32.0 ± 6.1	32.0 ± 6.0	32.3 ± 6.6
< 25	1,667 (8.4	1,227 (8.2)	440 (9.2)
25-< 30	6,565 (33.2)	5,031 (33.6)	1,534 (32.0)
30-< 35	6,305 (31.9)	4,850 (32.4)	1,455 (30.3)
≥ 35	5,241 (26.5)	3,874 (25.9)	1,367 (28.5)
AHI, events/h	36.9 ± 22.1	38.3 ± 22.2	32.6 ± 21.3
< 5	202 (1.0)	118 (0.8)	84 (1.7)
5-< 15	2,491 (12.3)	1,579 (10.3)	912 (18.5)
15-< 30	6,287 (31.1)	4,652 (30.4)	1,635 (33.1)
≥ 30	11,252 (55.6)	8,944 (58.5)	2,308 (46.7)
ESS	10.4 ± 5.0	10.5 ± 4.9	9.9 ± 5.0
< 7	4,557 (24,3)	3,309 (23.2)	1,248 (27.8)
7-10	5,118 (27.3)	3,859 (27.1)	1,259 (28.0)
11-15	5,982 (31.9)	4,669 (32.8)	1,313 (29.2)
> 16	3,072 (16.4)	2,396 (16.8)	676 (15.0)
Use of humidifier	10,028 (49.3)	7,686 (49.9)	2,342 (47.4)
Civil status			
Unmarried	4,736 (23.1)	3,366 (21.7)	1,370 (27.4)
Married	11,509 (56.1)	9,046 (58.4)	2,462 (49.3)
Divorced	3,319 (16.29	2,396 (15.5)	923 (18.5)
Widower or widow	935 (4.6)	696 (4.5)	239 (4.8)
Educational level			
Low, ≤ 9 y	4,392 (22.2)	3,256 (21.7)	1,140 (23.9)
Medium, 10-12 y	10,222 (51.7)	7,669 (51.1)	2,550 (53.5)
High, > 12 y	5,168 (26.1)	4,088 (27.2)	1,082 (22.6)
Households total income, index-linked gross pay, €	32,270 ± 18,921	32,861 ± 19,056	30,437 ± 18,540
1st quartile (lowest)	5,129 (25.0)	3,674 (23.7)	1,455 (29.1)
2nd quartile	5,128 (25.0)	3,853 (24.8)	1,275 (25.5)
3rd quartile	5,130 (25.0)	3,905 (25.2)	1,225 (24.5)
4th quartile (highest)	5,128 (25.0)	4,078 (26.3)	1,050 (21.0)
Birth country			
Born abroad	2,335 (11.4)	1,617 (10.4)	718 (14.3)
Born in Sweden, two foreign parents	427 (2.1)	298 (1.9)	129 (2.6)
Born in Sweden, one native parent	1,149 (5.6)	864 (5.6)	285 (5.7)
Born in Sweden, two native parents	16,610 (80.9)	12,732 (82.1)	3,878 (77.4)

Data are presented as No. (%), mean ± SD, or median (interquartile range). AHI = apnea-hypopnea index; ESS = Epworth Sleepiness Scale.

**Figure 3:**
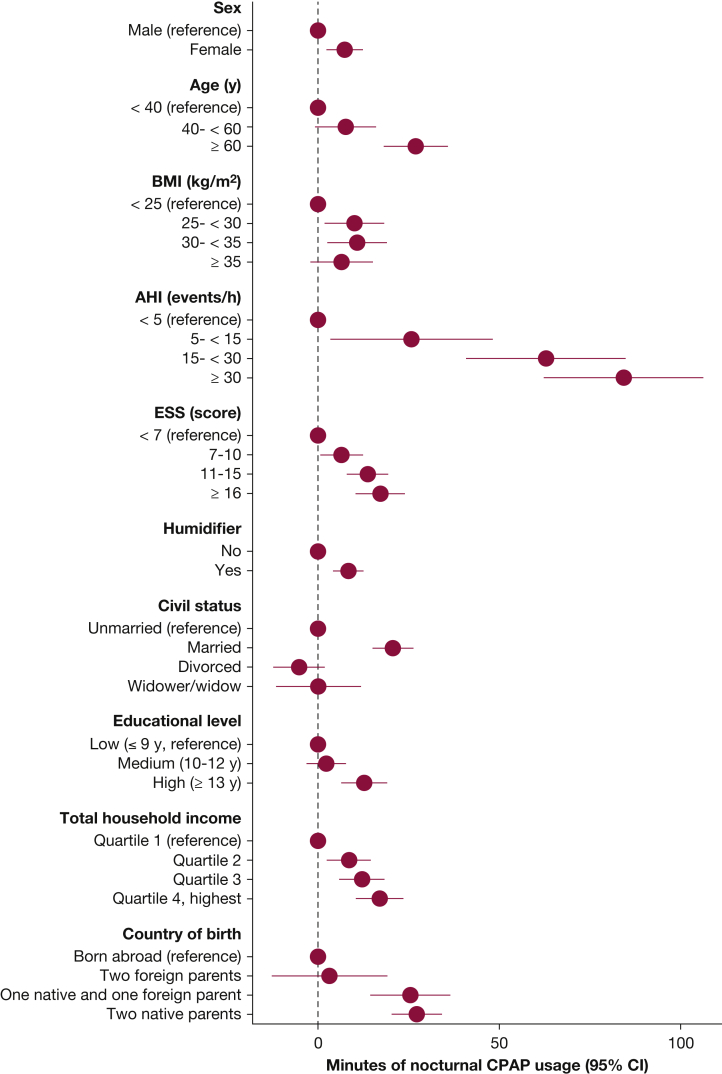
Forest plot showing the contribution of independent variables on total nocturnal CPAP use (min/night). AHI = apnea-hypopnea index; ESS = Epworth Sleepiness Scale.

In multivariate linear regression analysis (Table 2), independent predictors of higher nocturnal CPAP use were female sex, age of ≥ 60 years, BMI of 25 to 35 kg/m^2^, AHI of ≥ 15 events/h, ESS of > 10, and use of a humidifier. Among the socioeconomic factors, being married, having a high educational level exceeding 13 years, having a total household income exceeding the lowest quartile, and being born in Sweden with one or two native parents all were associated independently with longer nocturnal CPAP use (Table 2, Fig 3).

**Table 2:** Multiple Linear Regression Models With Minutes of Nightly CPAP Use as Dependent Variable

Variable	Model 2A: β-Coefficient for Minutes of Nightly CPAP Use (95% CI)^a^	*P* Value	Model 2B: β-Coefficient for Minutes of Nightly CPAP Use (95% CI)^b^	*P* Value	Model 2C: β-Coefficient for Minutes of Nightly CPAP Use (95% CI)^c^	*P* Value
Sex						
Male	1	...	1	...	1	...
Female	–1.7 (–6.2 to 2.8)	.460	3.1 (–1.7 to 7.9)	.208	7.3 (2.3-12.4)	.005
Age, y						
< 40	1	...	1	...	1	...
40-60	13.6 (5.7-21.4)	.001	7.5 (–0.8 to 15.7)	.076	7.6 (–0.9 to 16.2)	.079
≥ 60	33.8 (26.0-41.6)	< .001	26.3 (17.6-34.9)	< .001	27.0 (18.0-36.0)	< .001
BMI, kg/m^2^						
< 25	...	...	...	...	1	...
25-< 30	...	...	...	...	10.0 (1.7-18.4)	.018
30-< 35	...	...	...	...	10.8 (2.3-19.2)	.012
≥ 35	...	...	...	...	6.5 (–2.3 to 15.2)	.146
AHI, events/h						
< 5	...	...	...	...	1	...
5-< 15	...	...	...	...	25.8 (3.2-48.4)	.025
15-< 30	...	...	...	...	62.9 (40.8-85.0)	< .001
≥ 30	...	...	...	...	84.4 (62.4-106.4)	< .001
ESS score	...	...	...	...	...	...
< 7	...	...	...	...	1	...
7-10	...	...	...	...	6.5 (0.5-12.5)	.032
11-15	...	...	...	...	13.7 (7.9-19.5)	< .001
> 16	...	...	...	...	17.2 (10.3-24.1)	< .001
Use of humidifier	...	...	...	...	8.4 (4.1-12.7)	< .001
Civil status						
Unmarried	...	...	1	...	1	...
Married	...	...	20.5 (14.8-25.6)	< .001	20.6 (14.9-26.3)	< .001
Divorced	...	...	–6.8 (–13.7 to 0.2)	.056	–5.2 (–12.4 to 2.0)	.415
Widower or widow	...	...	3.9 (–7.2 to 15.1)	.489	0.1 (–11.6 to 11.8)	.984
Educational level						
Low (≤ 9 y)	...	...	1	...	1	...
Medium (10-12 y)	...	...	3.1 (–2.2 to 8.4)	.256	2.3 (–3.2 to 7.9)	.415
High (≥ 13 y)	...	...	13.2 (7.0-19.4)	< .001	12.8 (6.3-19.2)	< .001
Household total income (index-linked)						
Quartile 1 (lowest income)	...	...	1	...	1	...
Quartile 2	...	...	6.1 (0.2-12.0)	.043	8.5 (2.3-14.6)	.007
Quartile 3	...	...	10.4 (4.3-16.5)	.001	12.1 (5.8-18.5)	< .001
Quartile 4 (highest income)	...	...	15.9 (9.5-22.3)	< .001	17.0 (10.3-23.7)	< .001
Birth country						
Born abroad	...	...	1	...	1	...
Born in Sweden, two foreign parents	...	...	10.7 (–4.9 to 26.3)	.179	3.2 (–12.9 to 19.3)	.698
Born in Sweden, one native parent	...	...	29.0 (18.3-39.8)	< .001	25.5 (14.4-36.6)	< .001
Born in Sweden, two native parents	...	...	29.3 (22.7-36.0)	< .001	27.2 (20.2-34.2)	< .001

AHI = apnea-hypopnea index; ESS = Epworth Sleepiness Scale.

a Adjusted for sex and age.

b Adjusted for sex, age, and socioeconomic factors.

c Adjusted for all variables in the table.

In a sensitivity analysis addressing a potential reporting bias, all socioeconomic factors remained significant as independent predictors of CPAP adherence in counties with more than 50% of patients with reported follow-up data (8 counties; 13,138 patients). In counties with less than 50% of patients with a reported follow-up visit (11 counties; 7,383 patients), all factors except education level and total household income for counties with < 50% follow-up remained significant (e-Table 1). The impact of socioeconomic factors on adherence to CPAP treatment was confirmed in a multivariate logistic regression analysis adjusting for the same covariates (Table 3). Patients lost to follow-up (n = 39,949) showed a slightly lower AHI at baseline compared with the analysis population (34.6 ± 22.7 vs 36.9 ± 22.1 events/h; *P* < .001); otherwise, no clinically significant differences at baseline were identified (e-Table 2).

**Table 3:** OR for Having Nocturnal CPAP Use of ≥ 4 h

Variable	OR (95% CI)	*P* Value
Civil status		
Unmarried	1	...
Married	1.36 (1.25-1.48)	< .001
Divorced	0.96 (0.87-1.07)	.498
Widower or widow	1.06 (0.88-1.27)	.547
Education		
Low (≤ 9 y)	1	...
Medium (10-12 y)	1.08 (0.99-1.17)	.079
High (≥13 y)	1.26 (1.14-1.39)	< .001
Household total income		
Quartile 1 (lowest income)	1	...
Quartile 2	1.15 (1.05-1.26)	.004
Quartile 3	1.26 (1.14-1.38)	< .001
Quartile 4 (highest income)	1.43 (1.29-1.59)	< .001
Birth country		
Born abroad	1	...
Born in Sweden, two foreign parents	1.09 (0.86-1.38)	.476
Born in Sweden, one native parent	1.38 (1.17-1.63)	< .001
Born in Sweden, two native parents	1.37 (1.24-1.52)	< .001

Adjusted for sex, age, and all variables in the table. AHI = apnea-hypopnea index; ESS = Epworth Sleepiness Scale.

## Discussion

The main finding of this longitudinal population-based study is that civil status, educational level, household income, and foreign background can be established as strong predictors for CPAP adherence in OSA. As illustrated in the regression analysis, the effect sizes of socioeconomic factors are equivalent to those often used for the indication of CPAP therapy like the degree of daytime sleepiness or OSA severity.

To the best of our knowledge, the impact of socioeconomic factors on adherence to CPAP therapy have not been analyzed in a large population-based cohort. Previous studies are based on small clinical cohorts consisting of 70 to 330 patients,^15^, ^16^, ^17^, ^18^, ^19^, ^20^, ^21^, ^22^ and follow-up times generally were short.^20^ Because of the small sample size in previous studies, multivariate analysis is statistically challenging, rendering the results inconsistent. To estimate the impact of socioeconomic status despite small sample sizes, different compound socioeconomic variables were created. Such compound variables were able to show associations with CPAP adherence in some studies,^17^ whereas others studies failed to do so.^20^^,^^21^ Indeed, neighborhood social status has been used as a proxy for socioeconomic factors, and some studies have found associations with CPAP adherence,^16^, ^17^, ^18^ whereas others have not.^23^

In the present study, being married was associated with longer time undergoing CPAP therapy. In a retrospective cohort study with 330 OSA patients, being married was associated with higher nocturnal CPAP use of ≥ 4 h after 1 week.^18^ In another study with 80 patients and a follow-up time of 1 month, patients living with a partner evinced higher CPAP use.^19^ Other studies have failed to show associations between civil status and CPAP adherence.^21^^,^^22^ Studies analyzing associations between educational level and CPAP adherence are sparse and the results are conflicting.^17^^,^^21^ Two studies from Israel and one from Iran have found associations with low income levels and impaired adherence^15^^,^^16^^,^^31^ and have pointed to patients’ inability to afford a CPAP device as a partial explanation. In Sweden, the health care system largely is tax funded, making patients mainly independent of private economy or private health care insurance to obtain access to CPAP treatment. In the present study, those with the lowest household total income showed lower CPAP use, a finding that accords with small studies from New Zealand and the United States, where low incomes were associated with less time using CPAP.^17^^,^^32^ In several studies from the United States and New Zealand, being Black or of non-European origin, respectively, were associated with worse adherence to CPAP therapy, but after adjusting for socioeconomic factors, the associations weakened or disappeared.^17^^,^^18^^,^^22^^,^^32^ In the present study, being born abroad or being born in Sweden with two foreign parents was associated independently with lower CPAP use. This may be explained by linguistic difficulties or cultural differences.

Mean AHI at initiation of CPAP therapy was 37, and adherence rate was 355 min of nocturnal CPAP use, both high rates compared with those reported from in other countries.^33^ In accordance with Swedish national guidelines, mild to moderate OSA often is treated with a mandibular advancement device, whereas severe OSA is treated preferably with CPAP. Presence of severe OSA is associated positively with adherence,^25^ and these factors together can explain, at least in part, the elevated baseline AHI and the proportionally high amount of CPAP adherence reported in our study when compared with data published elsewhere.^13^^,^^33^ Age and degree of sleep apnea at baseline did not differ substantially between reporting centers. In contrast, partial copayment for CPAP therapy by the patients may vary substantially between administrative regions in Sweden (€0-€200 per year). However, mean CPAP adherence differs only to a small extent between different regions in Sweden (e-Fig 2).^34^

As expected, we identified significant intercorrelations between socioeconomic factors (eg, education level and income) as well as between socioeconomic factors and anthropometric factors (eg, education level and BMI). However, the influence of socioeconomic factors on compliance remained unchanged in the final statistical model (data not shown).

The current study has a number of strengths, including the generalizability of our findings. First, this large patient cohort has a nationwide coverage of more than 90% of all patients with OSA treated with CPAP in Sweden. In addition, reported Swedevox registry data have a high degree of internal and external validity, as evidenced by an actual validation study that demonstrated > 98% agreement of register data and source data in the medical records.^35^ Cross-linkage of our patient data with high-quality socioeconomic data from the nationwide Swedish Civil Registry and Tax Authorities creates a world-unique database in size and data quality.

A number of limitations need to be discussed, including a high proportion of patients lost to follow-up (more than 60%), which may have created an important bias in our analysis. Despite development and spread of remote monitoring of CPAP treatment, data still are reported to the Swedevox registry manually via a web questionnaire. Plenty of technological and juridical issues must be solved first, but automated follow-up reporting definitely would increase data capture and would strengthen overall data quality from the registry. Plausible explanations of low reported follow-up rates are that reporting to the registry is down-prioritized at some centers or that patients do not show up when called for control. In the current study, 76% of the patients showed nocturnal CPAP use exceeding 4 h at the 1-year follow-up visit, a proportion slightly higher compared with what was found in previous studies,^13^ supporting the assumption that patients not attending follow-up assessments may have worse adherence rates. We performed a number of additional analyses. First, we identified no clinically significant differences in baseline characteristics between included and not included patients, which reduces the risk of bias. In addition, our final study cohort was slightly biased toward a lower proportion of unmarried patients and those born abroad, two factors associated with lower compliance. Therefore, our analysis may rather underestimate the impact of these socioeconomic factors on CPAP adherence. Finally, a high rate of completed follow-up visits is a quality marker for generalizability of data, so we performed a sensitivity analysis including centers with high (> 50%) and low (< 50%) follow-up rates. In centers with lower follow-up frequency, educational level and total household income were no longer associated with adherence. Thus, this sensitivity analysis confirmed the strong influence of socioeconomic factors on CPAP adherence because the association between socioeconomic factors and adherence was even stronger in this subpopulation characterized by less selection bias.

Our results show that socioeconomic factors such as economic, educational, and cultural background affect CPAP treatment adherence. Low socioeconomic status is associated with poorer health and shorter life expectancy.^36^ Identifying modifiable factors like treatment adherence that could be part of the explanation for these differences—and acting on these findings—could contribute to better health. When treating patients with CPAP, a greater awareness of the impact of different socioeconomic factors on adherence and, when necessary, individually tailored follow-up may improve treatment adherence and may contribute to health equity.

## Interpretation

Civil status, educational level, household income, and foreign background are important factors associated with adherence to CPAP therapy in patients with OSA. To promote adherence, socioeconomic factors should be considered. Education and follow-up programs should be tailored better to people with low socioeconomic status.

**Take-home Points**

**Study Question:** How do socioeconomic factors influence CPAP adherence?

**Results:** Civil status, educational level, household income, and foreign background predict CPAP adherence in a clinically significant manner.

**Interpretation:** Education and follow-up programs should be tailored better to people with low socioeconomic status.

## Supplementary Data

e-Online Data
