# Supplementary material for: Socioeconomic Factors and Adherence to CPAP: The Population-Based Course of Disease in Patients Reported to the Swedish CPAP Oxygen and Ventilator Registry Study
Source: Chest. 2021 May 8;160(4):1481–91. doi: 10.1016/j.chest.2021.04.064 (PMC8546239; doi:10.1016/j.chest.2021.04.064)
Supplement: e-Online Data [file mmc1.pdf]

CHEST Online Supplement

Socioeconomic Factors and Adherence to CPAP

The Population-Based Course of Disease in Patients Reported to the Swedish CPAP Oxygen and Ventilator Registry Study

Andreas Palm, MD, PhD; Ludger Grote, MD, PhD; Jenny Theorell-Haglöw, PhD; Mirjam Ljunggren, MD, PhD; Josefin Sundh, MD, PhD; Bengt Midgren, MD, PhD; and Magnus Ekström, MD, PhD

CHEST 2021; 160(4):1481-1491

Online supplements are not copyedited prior to posting and the author(s) take full responsibility for the accuracy of all data.

© 2021 AMERICAN COLLEGE OF CHEST PHYSICIANS. Reproduction of this article is prohibited without written permission from the American College of Chest Physicians. See online for more details. **DOI:** 10.1016/j.chest.2021.04.064

e-Table 1. Sensitivity analysis. Multiple linear regression with minutes of nightly CPAP usage as dependent variable. Counties with < 50% and ≥50 % analyzed separately. Adjusted for age and sex and all variables in the table.

	β-Coefficient, minutes of nightly CPAP usage (95% CI). Centers with <50 follow-up-rate n=7,383	p-value	β-Coefficient, minutes of nightly CPAP usage (95% CI). Centres with ≥50 % follow-up-rate n=13,138	p-value
**Civil status**				
Unmarried	1		1	
Divorced	-5.6 (-16.2-4.9)	0.296	-5.5 (-14.5-3.6)	0.236
Widower/widow	6.1 (-10.8-23.0)	0.479	4.2 (-10.3-18.8)	0.568
Married	18.9 (10.6-27.1)	<0.001	23.2 (16.1-30.4)	<0.001
**Education**				
Low (≤9 years)	1		1	
Medium (10-12 years)	3.9 (-4.5-12.3)	0.360	2.1 (-4.7-8.9)	0.551
High (≥13 years)	8.4 (-1.0-17.8)	0.081	13.4 (5.4-21.5)	0.001
**Household total income** (Index-linked)				
Quartile 1 (lowest income)	1		1	
Quartile 2	2.9 (-6.4-12.1)	0.545	7.7 (0.1-15.2)	0.047
Quartile 3	8.2 (-1.3-17.7)	0.089	11.2 (3.4-19.1)	0.005
Quartile 4 (highest income	7.8 (-1.8-17.5)	0.112	17.8 (9.5-26.2)	<0.001
**Birth country**				
Born abroad n (%)	1		1	
Born in Sweden, two foreign parents n (%)	10.0 (-16.4-36.4)	0.459	14.9 (-4.3-34.2)	0.129
Born in Sweden, one native parent n (%)	31.3 (14.9-47.8)	<0.001	29.0 (15.2-42.9)	<0.001
Born in Sweden, two native parents n (%)	32.8 (22.6-42.9)	<0.001	28.2 (19.5-36.9)	<0.001

CI: Confidence interval; CPAP: Continuous positive airway pressure

Online supplements are not copyedited prior to posting and the author(s) take full responsibility for the accuracy of all data.

e-Table 2. Comparison of baseline characteristics between study population and those not registered for follow-up.

	Study population n=20,521	Lost to follow-up n=39,949	p-value
**Men n (%)**	14,501 (70.7)	27,990 (70.0)	0.126
**Age, years**	57.8±12.2	57.2±12.7)	<0.001
**BMI, kg/m2**	32.0±6.1	31.8±6.2	<0.001
**AHI, events/hour**	36.9±22.1	34.6±22.7	<0.001
**ESS, score**	10.4±5.0	10.2±5.0	0.001
**Use of humidifier n (%)**	10,028 (49.3)	19,274 (56.8)	<0.001
**Civil status**			
Unmarried n (%)	4,736 (23.1)	9,177 (24.4)	<0.001
Married n (%)	11,509 (56.1)	19,846 (52.8)	
Divorced n (%)	3,319 (16.2)	6,869 (18.3)	
Widower/widow n (%)	935 (4.6)	1,670 (4.5)	
**Level of education**			
Low (≤9 years)	4,395 (22.2)	5,845 (21.9)	0.060
Medium (10-12 years)	10,218 (51.7)	13,638(51.0)	
High (≥13 years)	5,168 (26.1)	7,246 (27.1)	
**Households total income** (Index-linked gross pay EURO)	32,270±18,921	31,834±20,825	0.0158
**Birth country**			
Born abroad n (%)	2,335 (11.4)	6,220 (15.6)	<0.001
Born in Sweden, two foreign parents n (%)	427 (2.1)	929 (2.3)	
Born in Sweden, one native parent n (%)	1,149 (5.6)	2,443 (6.1)	
Born in Sweden, two native parents n (%)	16,610 (80.9)	30,356 (76.0)	

AHI: apnea-hypopnea index; BMI: body mass index; ESS: Epworth Sleepiness Scale

Results are presented as n (%) for categorical variables, as mean ± SD for normal distributed continuous variables.

Online supplements are not copyedited prior to posting and the author(s) take full responsibility for the accuracy of all data.

e-Figure 1. Clinical units in Sweden reporting patients with CPAP to the Swedevox registry 2019.

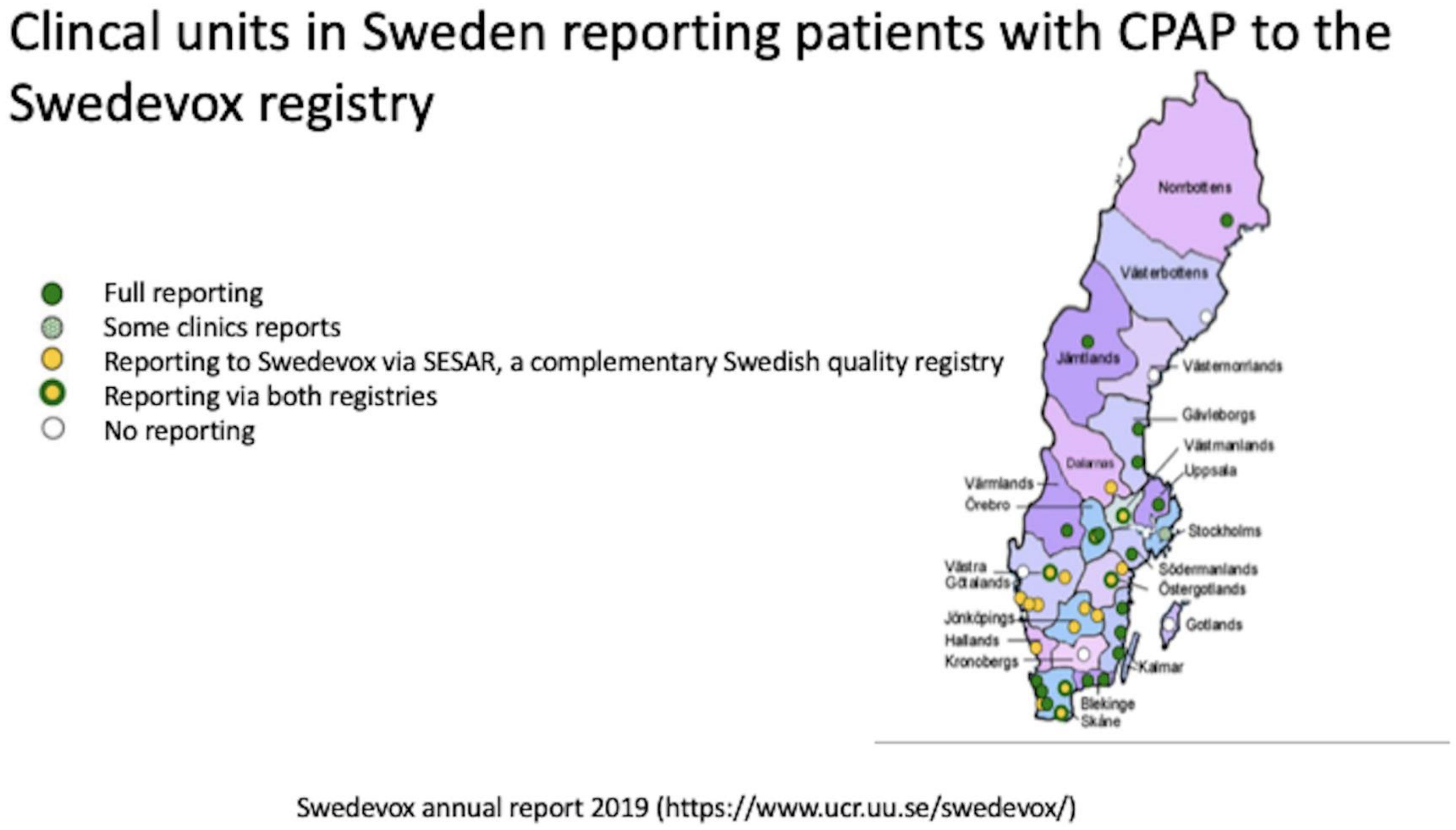

e-Figure 2. Mean age (a), mean AHI at CPAP initiation (b) and CPAP usage, mean hours/night (c), at 1-year follow-up 2019 for the different reporting sleep centers of the Swedish quality registry Swedevox registry. (Swedevox annual report 2019, https://www.ucr.uu.se/swedevox/.)

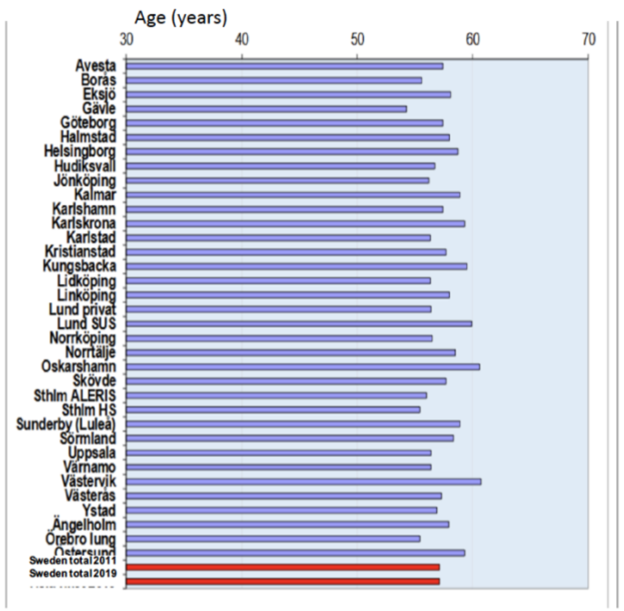

Supplemental Figure S2a

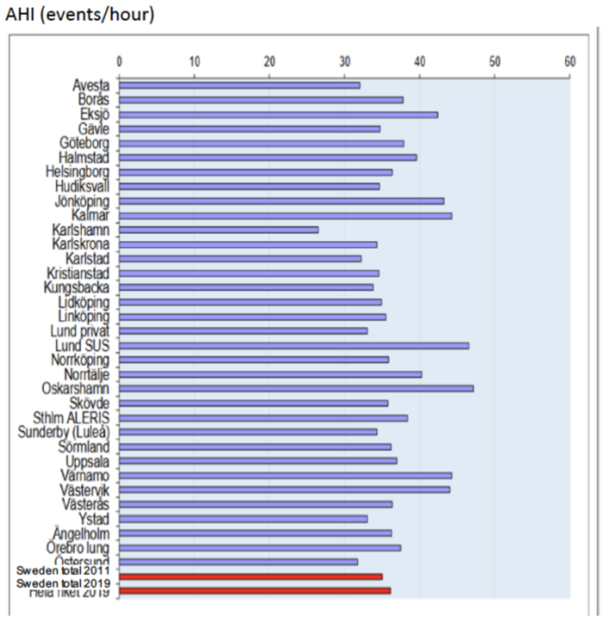

Supplemental Figure S2b

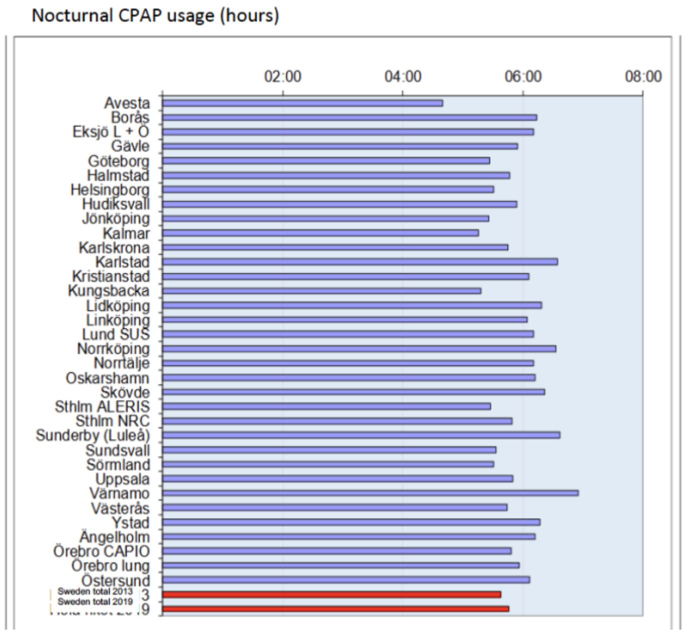

Supplemental Figure S2c

Swedevox annual report 2019 (https://www.ucr.uu.se/swedevox/)

AHI: apnea-hypopnoea index; CPAP: continuous positive airway pressure

205706

Online supplements are not copyedited prior to posting and the author(s) take full responsibility for the accuracy of all data.
